# Supplementary material for: A deep reinforcement learning platform for antibiotic discovery
Source: bioRxiv. 2025 Sep 23:2025.09.23.678086. Preprint. [Version 1] doi: 10.1101/2025.09.23.678086 (PMC12485834; doi:10.1101/2025.09.23.678086)
Supplement: Supplement 1 [file NIHPP2025.09.23.678086v1-supplement-1.pdf]

bioRxiv preprint doi: https://doi.org/10.1101/2025.09.23.678086; this version posted September 23, 2025. The copyright holder for this preprint (which was not certified by peer review) is the author/funder, who has granted bioRxiv a license to display the preprint in perpetuity. It is made available under aCC-BY 4.0 International license.

Supplementary Information

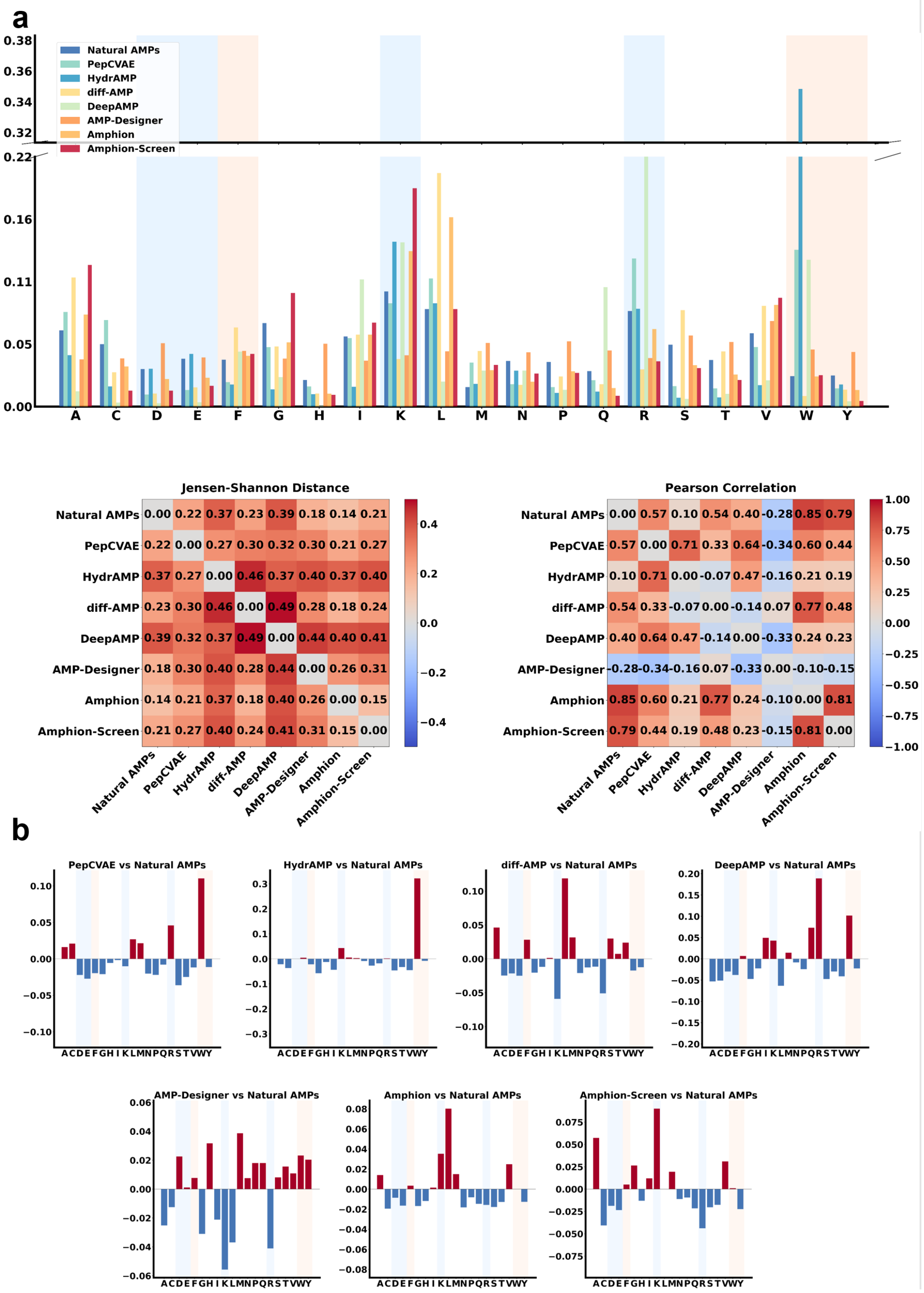

Supplementary Figure 1. Supplementary experiments on ApexAmphion's Generation. a) The amino acid frequency distribution between Amphion's and other baselines' samples and the natural AMPs. **b)** The difference of amino acid frequency distribution between Amphion's and other baselines' samples and the natural AMPs.

bioRxiv preprint doi: https://doi.org/10.1101/2025.09.23.678086; this version posted September 23, 2025. The copyright holder for this preprint (which was not certified by peer review) is the author/funder, who has granted bioRxiv a license to display the preprint in perpetuity. It is made available under aCC-BY 4.0 International license.

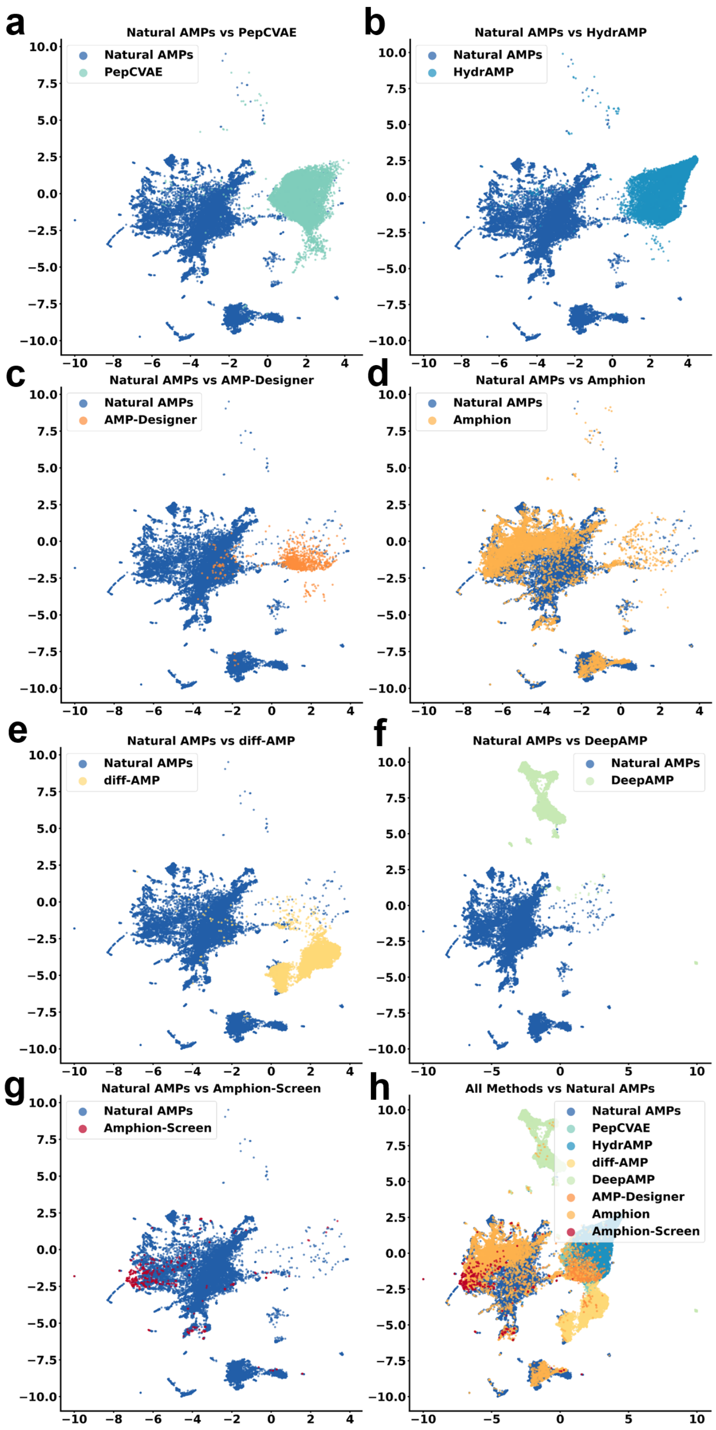

Supplementary Figure 2. Supplementary UMAP visualization of generated samples of AMP generation methods against natural AMPs. a-g) Visualization of PepCVAE, HydrAMP, AMP-Designer, diff-AMP, DeepAMP, Amphion, Amphion-Screen against natural AMPs. **h)** Combined plot of all methods and natural AMPs.

bioRxiv preprint doi: https://doi.org/10.1101/2025.09.23.678086; this version posted September 23, 2025. The copyright holder for this preprint (which was not certified by peer review) is the author/funder, who has granted bioRxiv a license to display the preprint in perpetuity. It is made available under aCC-BY 4.0 International license.

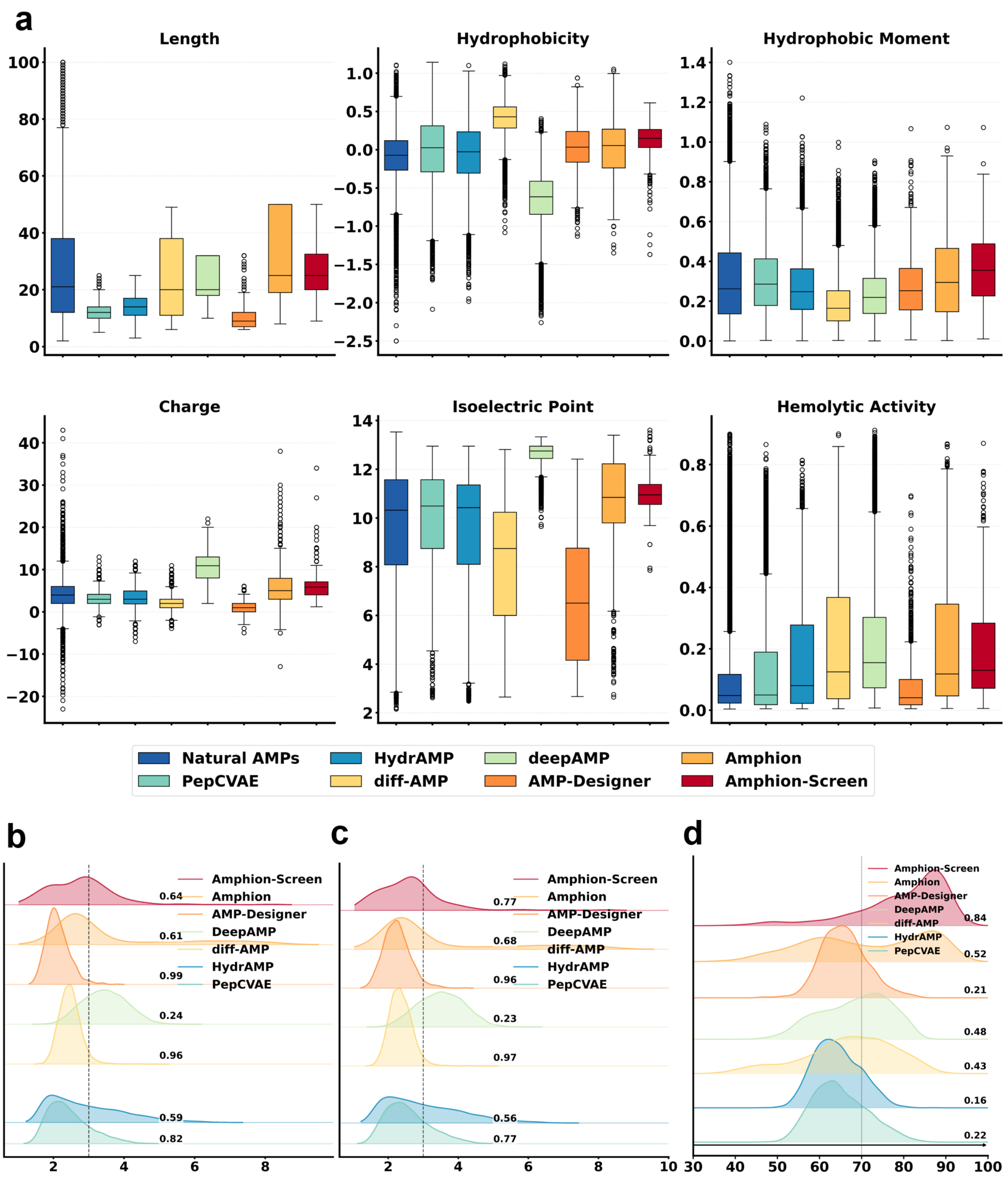

Supplementary Figure 3. Supplementary experiments on ApexAmphion's generation. a) The property distribution (Length, Hydrophobicity, Hydrophobic moment, Net charge, Isoeletric point, and Hemolytic activity) for all computational AMP generative methods. **b-d)** The latent distribution visualization under Amphion's samples against the other computational generative baselines according to the latent distance to Natural AMPs, the latent distance to natural low-MIC AMPs, and the pLDDT scores. The number on the right of each distribution denote the proportion under (B-C) and surpass (D) the thresholds.

bioRxiv preprint doi: https://doi.org/10.1101/2025.09.23.678086; this version posted September 23, 2025. The copyright holder for this preprint (which was not certified by peer review) is the author/funder, who has granted bioRxiv a license to display the preprint in perpetuity. It is made available under aCC-BY 4.0 International license.

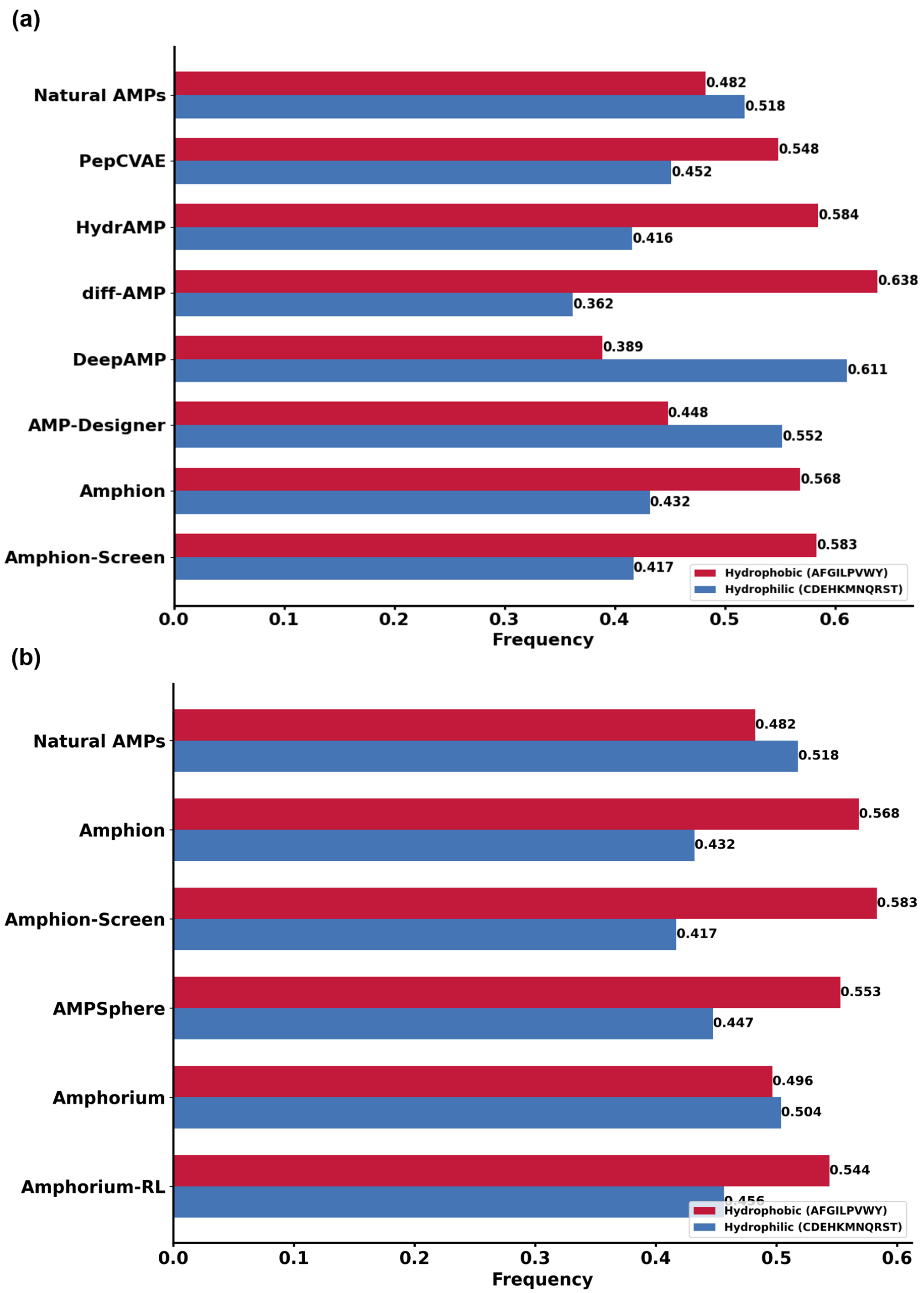

Supplementary Figure 4. Supplementary experiments on hydrophobic and hydrophilic amino acid frequency.a) The hydrophobic and hydrophilic amino acid frequency distribution among Amphion, Amphion-Screen, and other AMP generation methods against the natural AMPs.**b)** The hydrophobic and hydrophilic amino acid frequency distribution between Amphorium, Amphorium-RL, Amphion, Amphion-Screen, and AMPSphere against the natural AMPs.

bioRxiv preprint doi: https://doi.org/10.1101/2025.09.23.678086; this version posted September 23, 2025. The copyright holder for this preprint (which was not certified by peer review) is the author/funder, who has granted bioRxiv a license to display the preprint in perpetuity. It is made available under aCC-BY 4.0 International license.

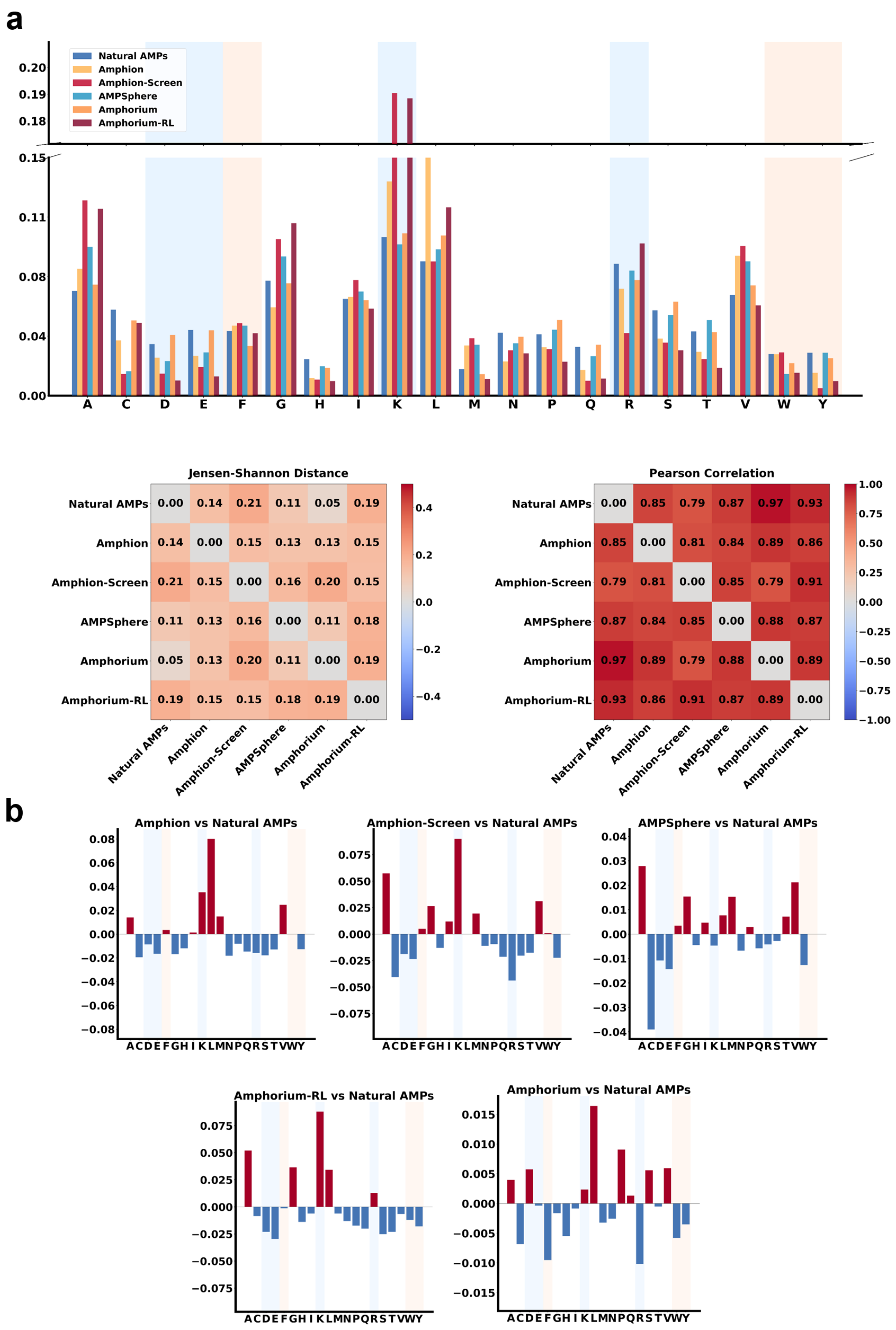

Supplementary Figure 5. Supplementary experiments on ApexAmphorium's entries. a) The amino acid frequency distribution between Amphorium, Amphorium-RL, Amphion, Amphion-Screen, and AMPSphere against the natural AMPs. **(b)** The difference of amino acid frequency distribution between Amphorium, Amphorium-RL, Amphion, Amphion-Screen, and AMPSphere against the natural AMPs.

bioRxiv preprint doi: https://doi.org/10.1101/2025.09.23.678086; this version posted September 23, 2025. The copyright holder for this preprint (which was not certified by peer review) is the author/funder, who has granted bioRxiv a license to display the preprint in perpetuity. It is made available under aCC-BY 4.0 International license.

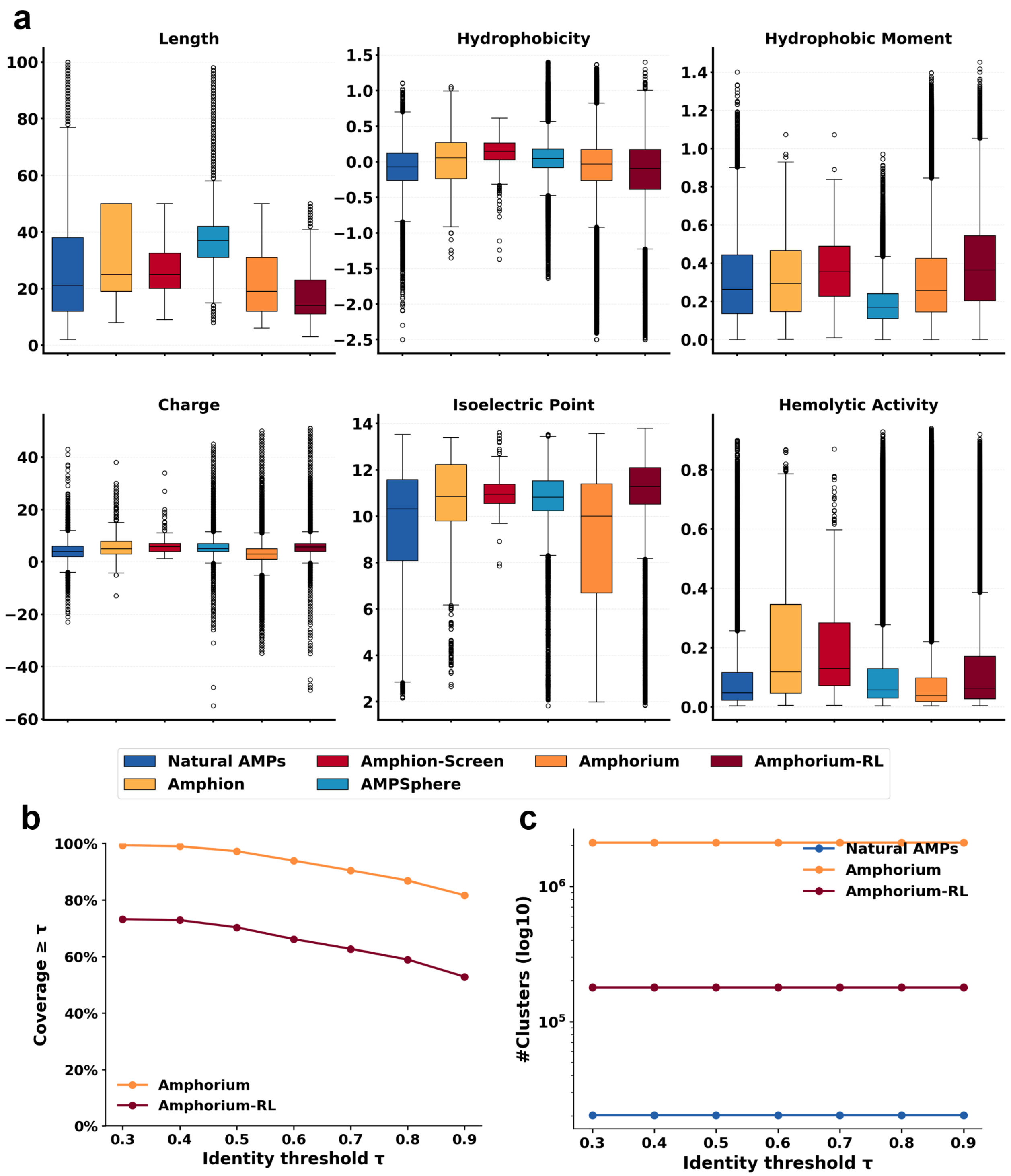

Supplementary Figure 6. Supplementary experiments on ApexAmphorium's entries. a) The property distribution (including length, hydrophobicity, hydrophobic moment, charge, isoeletric point, and hemolytic activity) between Amphorium, Amphorium-RL, Amphion, Amphion-Screen, and AMPSphere against the natural AMPs. **b-c)** Novelty and diversity analysis of Amphorium against natural AMPs by MMseqs2.

bioRxiv preprint doi: https://doi.org/10.1101/2025.09.23.678086; this version posted September 23, 2025. The copyright holder for this preprint (which was not certified by peer review) is the author/funder, who has granted bioRxiv a license to display the preprint in perpetuity. It is made available under aCC-BY 4.0 International license.

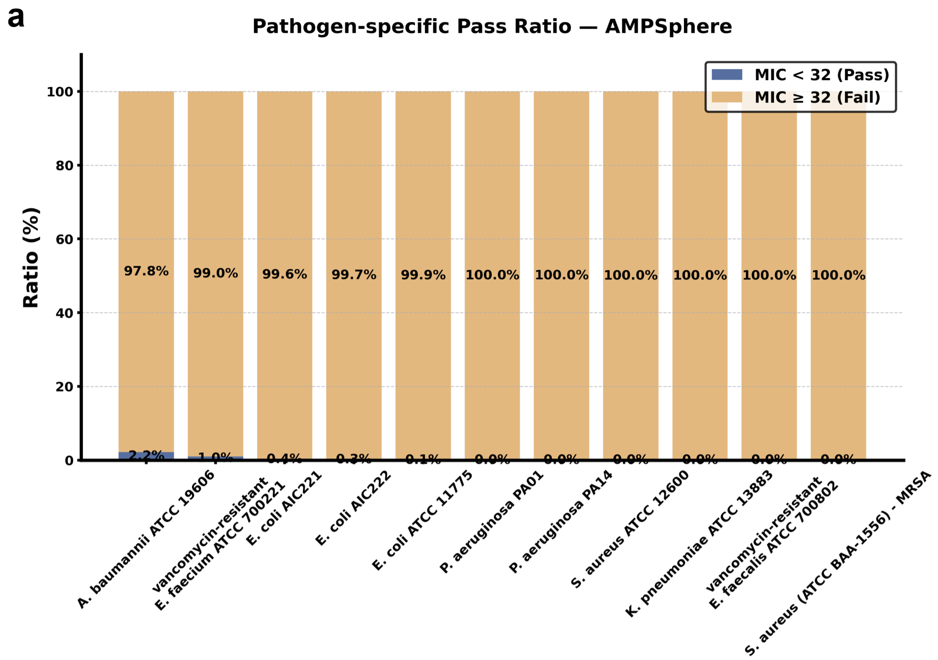

Pathogen	Pass Ratio	Pass Amount	Fail Amount
A. baumannii ATCC 19606	2.2%	18533/836966	97.8%
vancomycin-resistant E. faecium ATCC 700221	1.0%	8661/836966	99.0%
E. coli AIC221	0.4%	3652/836966	99.6%
E. coli AIC222	0.3%	2363/836966	99.7%
E. coli ATCC 11775	0.1%	559/836966	99.9%
P. aeruginosa PA01	0.0%	271/836966	100.0%
P. aeruginosa PA14	0.0%	225/836966	100.0%
S. aureus ATCC 12600	0.0%	9/836966	100.0%
K. pneumoniae ATCC 13883	0.0%	1/836966	100.0%
vancomycin-resistant E. faecalis ATCC 700802	0.0%	1/836966	100.0%
S. aureus (ATCC BAA-1556) - MRSA	0.0%	0/836966	100.0%

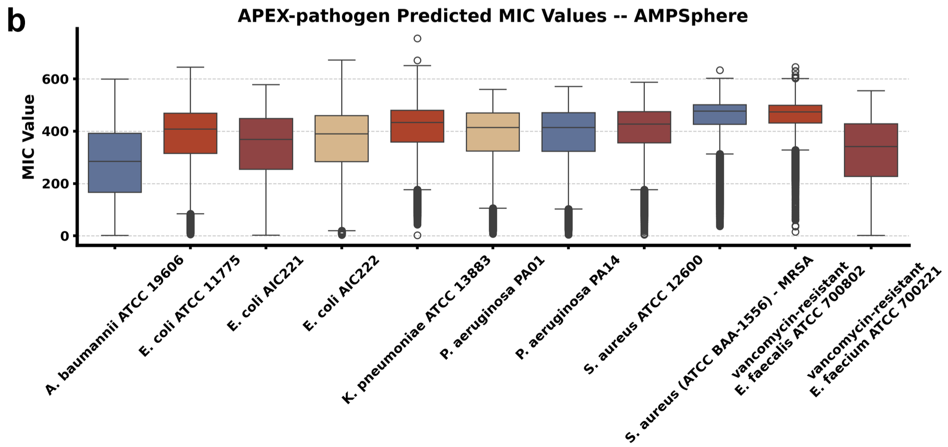

Supplementary Figure 7. Supplementary results of Apex 1.1 annotated distribution on AMPSphere entries. a) The pass ratio of AMPSphere entries on each pathogens under the condition of MIC <32 μmol L^{-1}. **b)** The predicted MIC value distribution of AMPSphere entries on each pathogens.

bioRxiv preprint doi: https://doi.org/10.1101/2025.09.23.678086; this version posted September 23, 2025. The copyright holder for this preprint (which was not certified by peer review) is the author/funder, who has granted bioRxiv a license to display the preprint in perpetuity. It is made available under aCC-BY 4.0 International license.

a

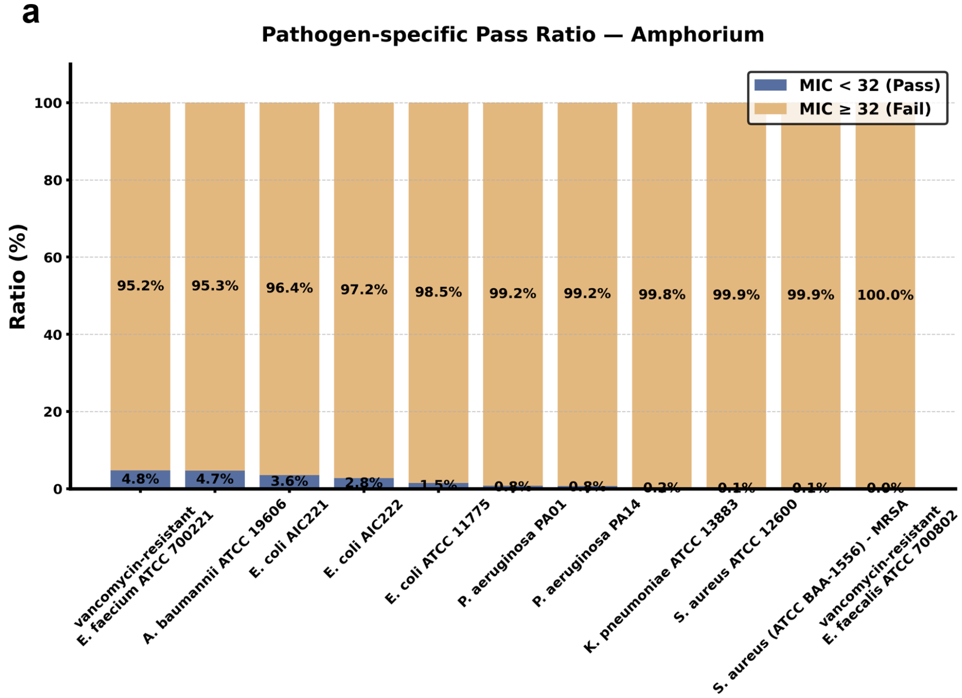

Pathogen	Pass Ratio	Pass Amount	Fail Amount
vancomycin-resistant E. faecium ATCC 700221	4.8%	100019/2097399	95.2%
A. baumannii ATCC 19606	4.7%	98524/2097399	95.3%
E. coli AIC221	3.6%	75187/2097399	96.4%
E. coli AIC222	2.8%	58117/2097399	97.2%
E. coli ATCC 11775	1.5%	31788/2097399	98.5%
P. aeruginosa PA01	0.8%	16001/2097399	99.2%
P. aeruginosa PA14	0.8%	15771/2097399	99.2%
K. pneumoniae ATCC 13883	0.2%	4465/2097399	99.8%
S. aureus ATCC 12600	0.1%	2365/2097399	99.9%
S. aureus (ATCC BAA-1556) - MRSA	0.1%	1953/2097399	99.9%
vancomycin-resistant E. faecalis ATCC 700802	0.0%	11/2097399	100.0%

b

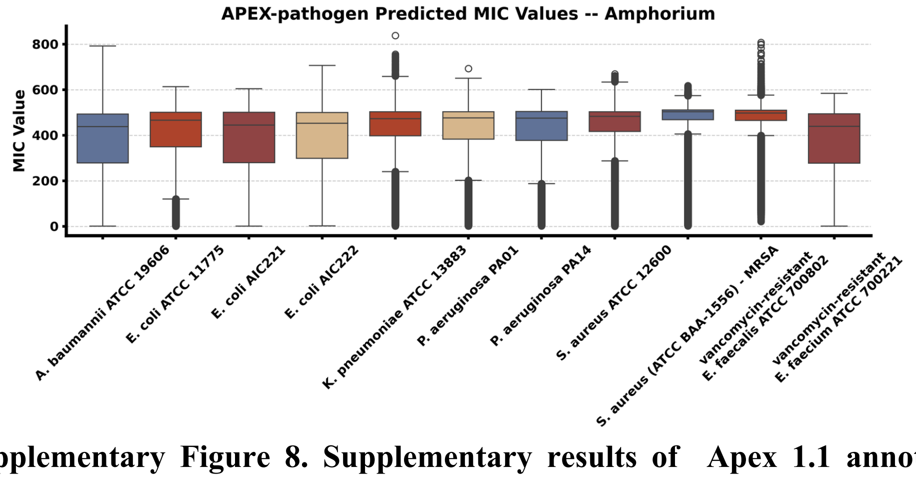

Supplementary Figure 8. Supplementary results of Apex 1.1 annotated distribution on ApexAmphorium entries. a) The pass ratio of ApexAmphorium entries on each pathogens under the condition of MIC <32 μmol L^{-1}. **b)** The predicted MIC value distribution of ApexAmphorium entries on each pathogens.

bioRxiv preprint doi: https://doi.org/10.1101/2025.09.23.678086; this version posted September 23, 2025. The copyright holder for this preprint (which was not certified by peer review) is the author/funder, who has granted bioRxiv a license to display the preprint in perpetuity. It is made available under aCC-BY 4.0 International license.

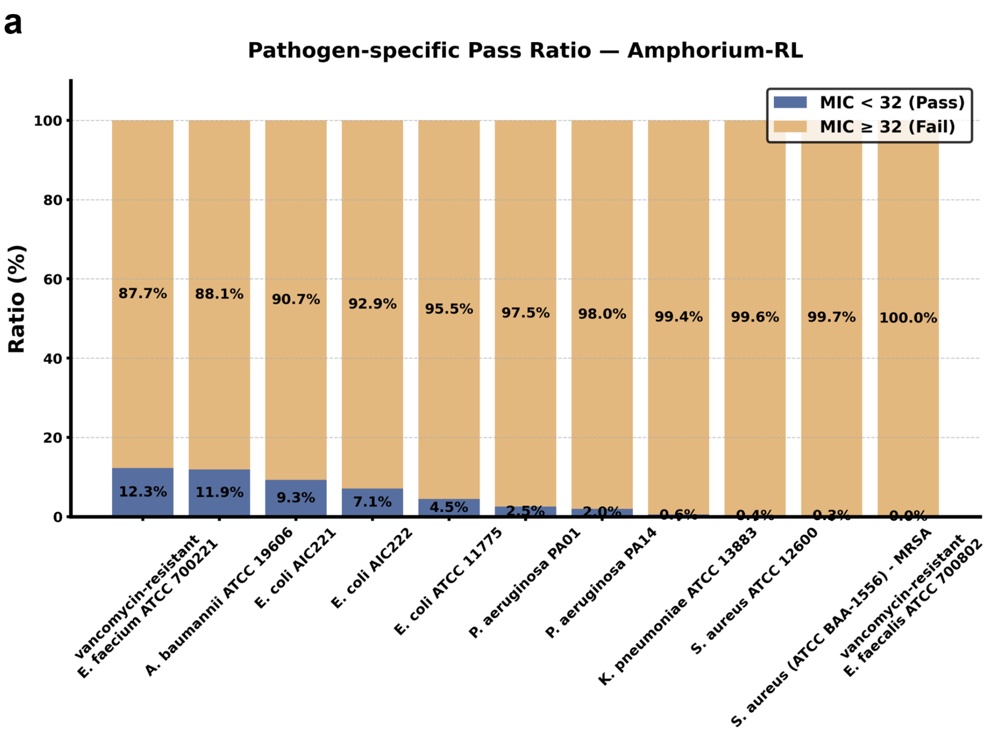

Pathogen	Pass Ratio	Pass Amount	Fail Amount
vancomycin-resistant E. faecium ATCC 700221	12.3%	22069/179600	87.7%
A. baumannii ATCC 19606	11.9%	21439/179600	88.1%
E. coli AIC221	9.3%	16720/179600	90.7%
E. coli AIC222	7.1%	12750/179600	92.9%
E. coli ATCC 11775	4.5%	7996/179600	95.5%
P. aeruginosa PA01	2.5%	4545/179600	97.5%
P. aeruginosa PA14	2.0%	3561/179600	98.0%
K. pneumoniae ATCC 13883	0.6%	1033/179600	99.4%
S. aureus ATCC 12600	0.4%	696/179600	99.6%
S. aureus (ATCC BAA-1556) - MRSA	0.3%	549/179600	99.7%
vancomycin-resistant E. faecalis ATCC 700802	0.0%	0/179600	100.0%

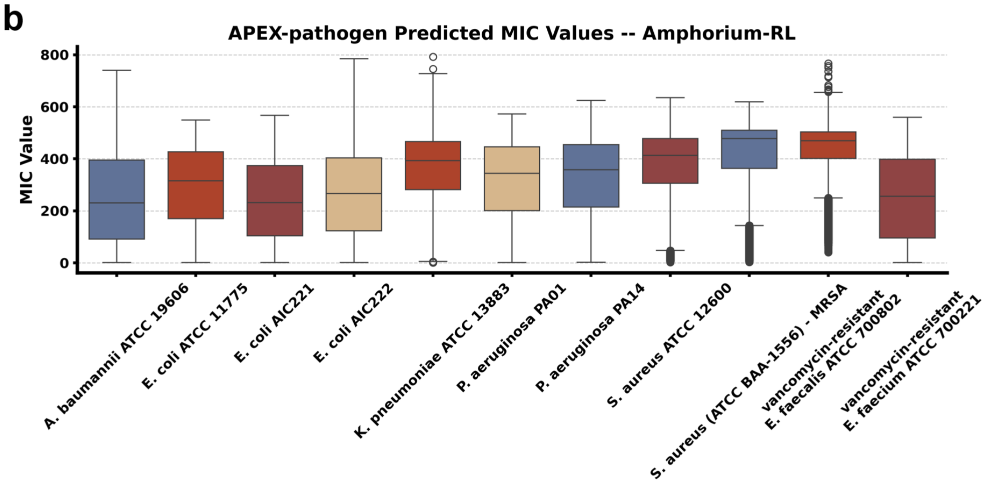

Supplementary Figure 9. Results of Apex 1.1 annotated distribution on ApexAmphorium-RL's entries. a) The pass ratio of ApexAmphorium-RL entries on each pathogens under the condition of MIC <32 μmol L^{-1}. **b)** The predicted MIC value distribution of ApexAmphorium-RL entries on each pathogens.

bioRxiv preprint doi: https://doi.org/10.1101/2025.09.23.678086; this version posted September 23, 2025. The copyright holder for this preprint (which was not certified by peer review) is the author/funder, who has granted bioRxiv a license to display the preprint in perpetuity. It is made available under aCC-BY 4.0 International license.

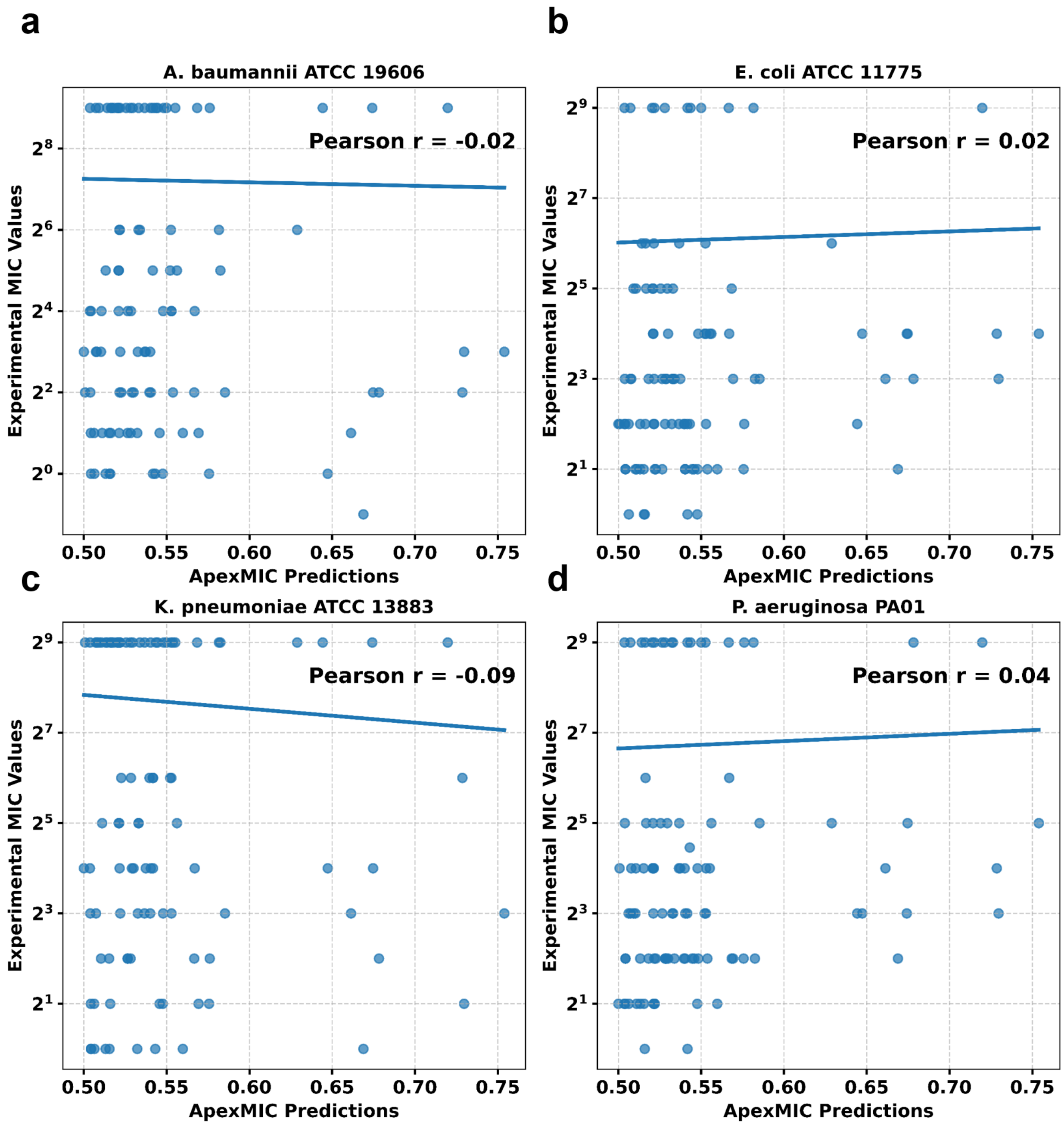

Supplementary Figure 10. Correlation analysis between ApexMIC's prediction and wet-lab experimental MIC values of amphionins. a-d) Sub-correlation between ApexMIC's predicted scores and tested MICs on different pathogens.

bioRxiv preprint doi: https://doi.org/10.1101/2025.09.23.678086; this version posted September 23, 2025. The copyright holder for this preprint (which was not certified by peer review) is the author/funder, who has granted bioRxiv a license to display the preprint in perpetuity. It is made available under aCC-BY 4.0 International license.

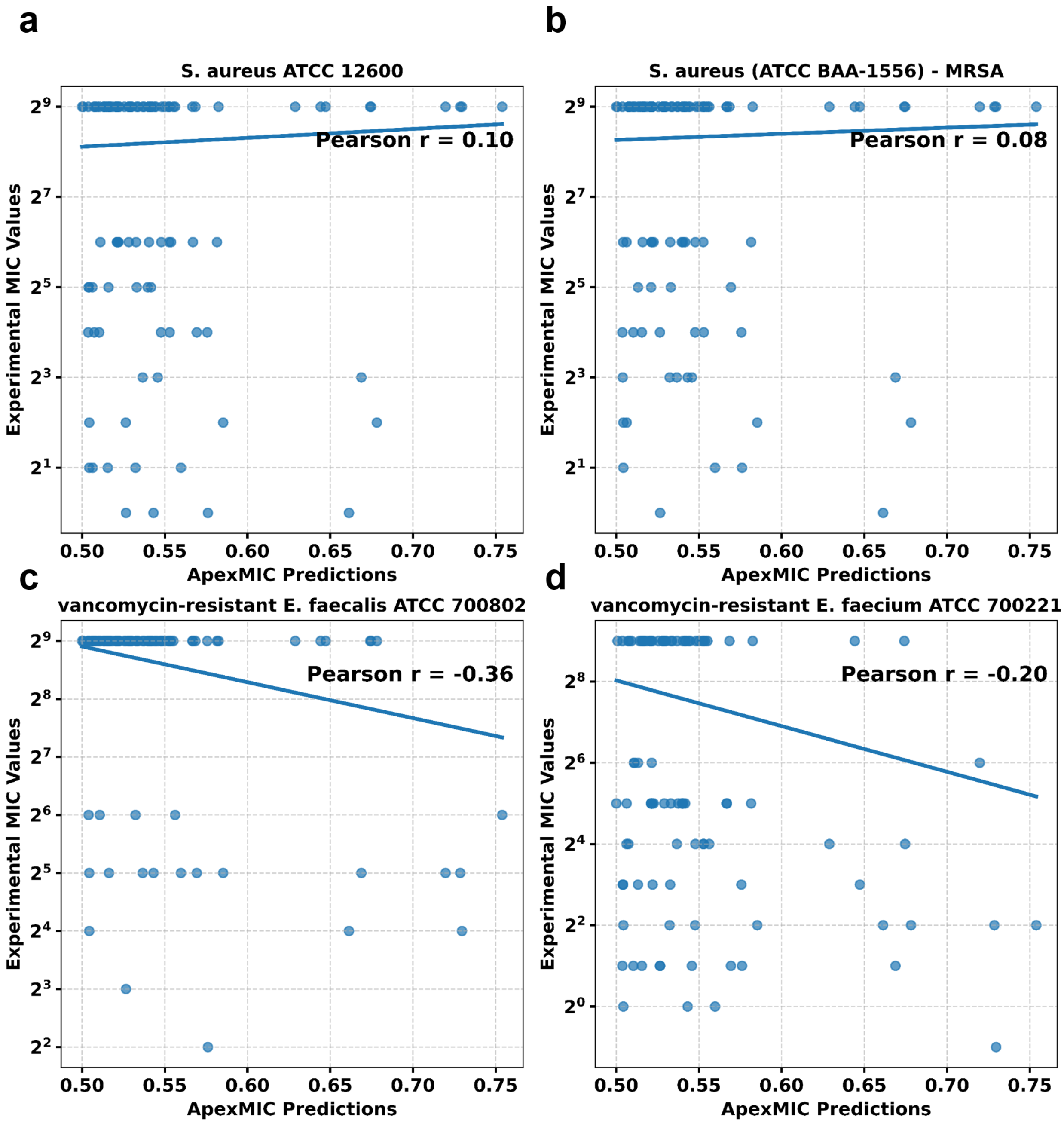

Supplementary Figure 11. Correlation analysis between ApexMIC's prediction and wet-lab experimental MIC values of amphionins. a-d) Sub-correlation between ApexMIC's predicted scores and tested MICs on different pathogens.

bioRxiv preprint doi: https://doi.org/10.1101/2025.09.23.678086; this version posted September 23, 2025. The copyright holder for this preprint (which was not certified by peer review) is the author/funder, who has granted bioRxiv a license to display the preprint in perpetuity. It is made available under aCC-BY 4.0 International license.

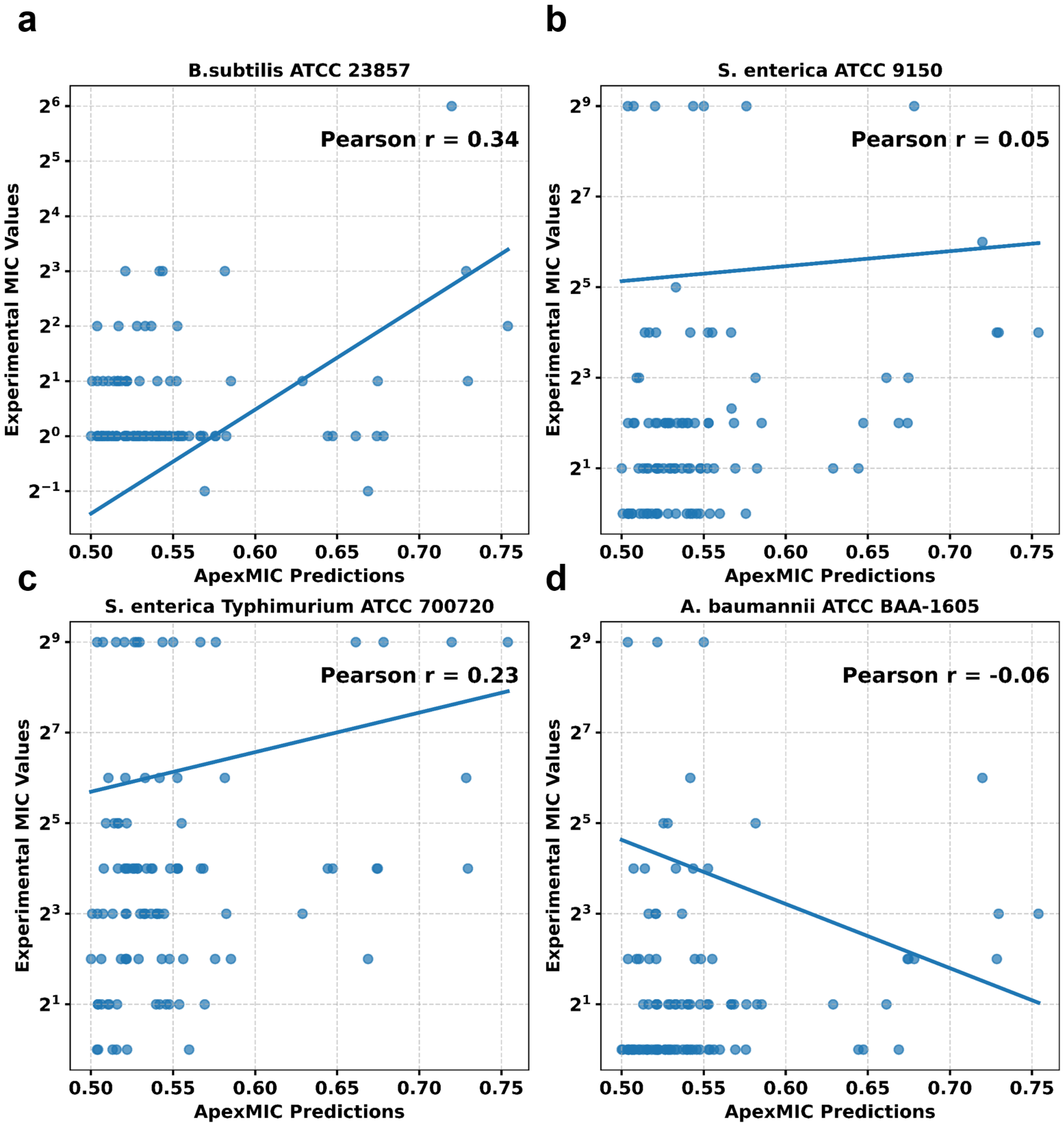

Supplementary Figure 12. Correlation analysis between ApexMIC's prediction and wet-lab experimental MIC values of amphionins. a-d) Sub-correlation between ApexMIC's predicted scores and tested MICs on different pathogens.

bioRxiv preprint doi: https://doi.org/10.1101/2025.09.23.678086; this version posted September 23, 2025. The copyright holder for this preprint (which was not certified by peer review) is the author/funder, who has granted bioRxiv a license to display the preprint in perpetuity. It is made available under aCC-BY 4.0 International license.

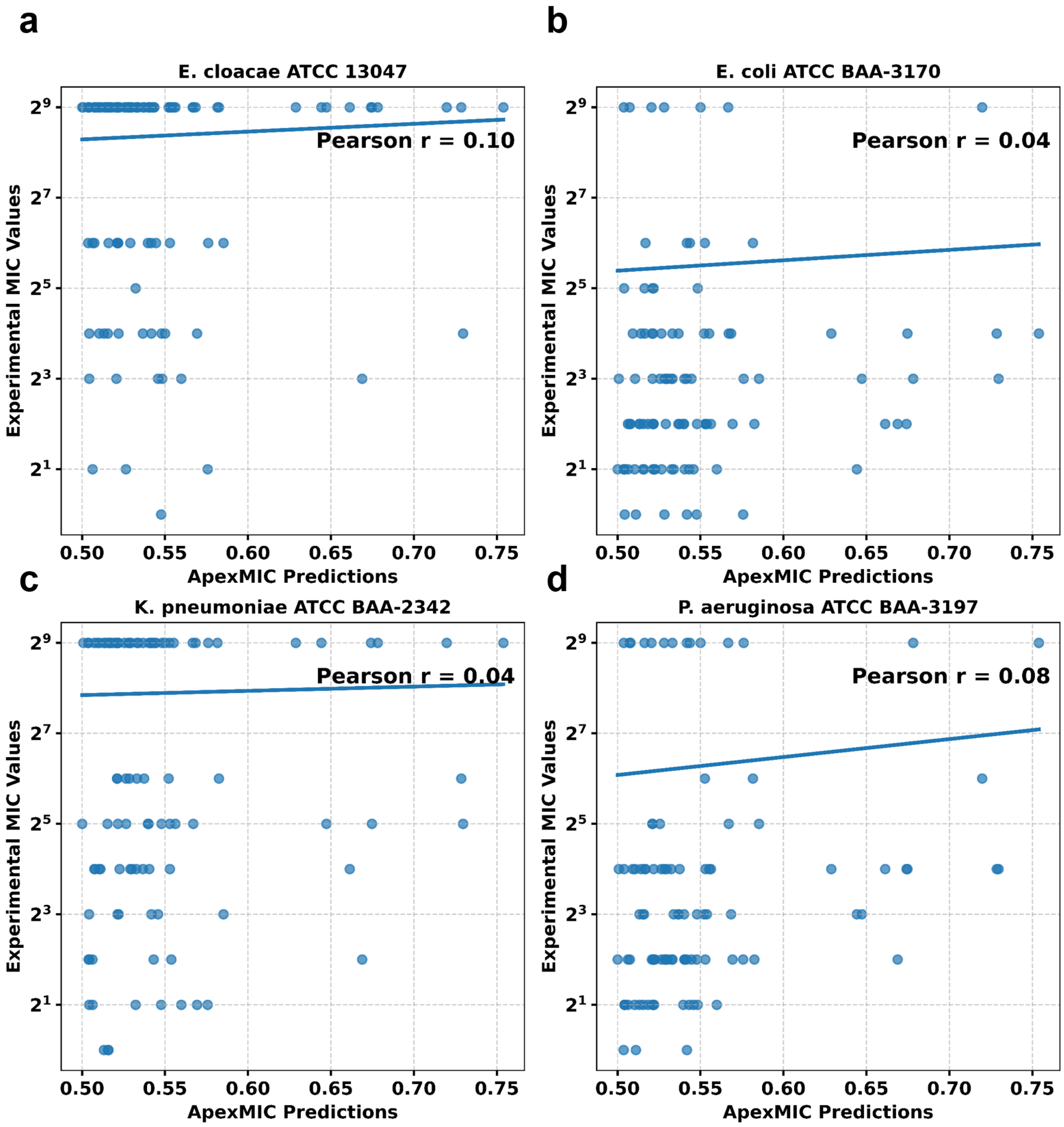

Supplementary Figure 13. Correlation analysis between ApexMIC's prediction and wet-lab experimental MIC values of amphionins. a-d) Sub-correlation between ApexMIC's predicted scores and tested MICs on different pathogens.
